# Real-Time 3D Single Particle Tracking: Towards Active Feedback Single Molecule Spectroscopy in Live Cells

**DOI:** 10.3390/molecules24152826

**Published:** 2019-08-02

**Authors:** Shangguo Hou, Courtney Johnson, Kevin Welsher

**Affiliations:** Department of Chemistry, Duke University, Durham, NC 27708, USA

**Keywords:** real-time 3D single particle tracking, single molecule spectroscopy, active feedback tracking

## Abstract

Single molecule fluorescence spectroscopy has been largely implemented using methods which require tethering of molecules to a substrate in order to make high temporal resolution measurements. However, the act of tethering a molecule requires that the molecule be removed from its environment. This is especially perturbative when measuring biomolecules such as enzymes, which may rely on the non-equilibrium and crowded cellular environment for normal function. A method which may be able to un-tether single molecule fluorescence spectroscopy is real-time 3D single particle tracking (RT-3D-SPT). RT-3D-SPT uses active feedback to effectively lock-on to freely diffusing particles so they can be measured continuously with up to photon-limited temporal resolution over large axial ranges. This review gives an overview of the various active feedback 3D single particle tracking methods, highlighting specialized detection and excitation schemes which enable high-speed real-time tracking. Furthermore, the combination of these active feedback methods with simultaneous live-cell imaging is discussed. Finally, the successes in real-time 3D single molecule tracking (RT-3D-SMT) thus far and the roadmap going forward for this promising family of techniques are discussed.

## 1. Introduction

**Figure 1:**
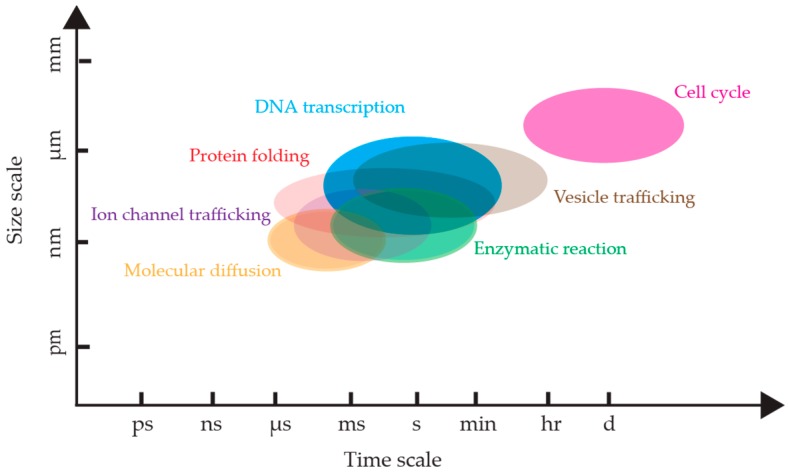
The temporal and spatial scales of biological processes. The time windows and size windows are approximate, and each process has its own situation-dependent scale. References: DNA transcription rate [29], protein folding [30,31,32], molecular diffusion [33,34,35], cell cycle [36], vesicle trafficking [37,38,39,40], ion channel trafficking [41,42,43], enzymatic reactions [3,44].

The advent of single molecule detection has led to a vast array of new scientific lines of inquiry [1,2,3]. Prior to the ability to measure molecules individually, individual molecular behaviors could only be deduced from the bulk, obscuring critical sub-populations and non-equilibrium dynamics. Bulk measurements are unable to untangle the fast motions of protein domains due to the asynchronous nature of the motions among different molecules in the ensemble. Observing molecules one at a time enables the direct measurement of these fast molecular motions which are critical to biological processes [4]. Single molecule measurements have shed light on a wide range of biomolecular dynamics including DNA repair [5], transcription [6,7,8], translation [9], RNA structure [10,11], nucleases [12], ribozyme catalysis [13,14], enzyme catalysis [3,15,16], intrinsically disordered proteins [17], and protein folding [18] (Figure 1). Single molecule measurements have also been the driving mechanisms behind the advent of single molecule based super-resolution methods. The various implementations of super-resolution microscopies have provided unprecedented spatial resolution down to several nanometers, enabling observation of the fine structure of cells in their native state [19,20,21,22,23,24,25,26,27,28].

**Figure 2:**
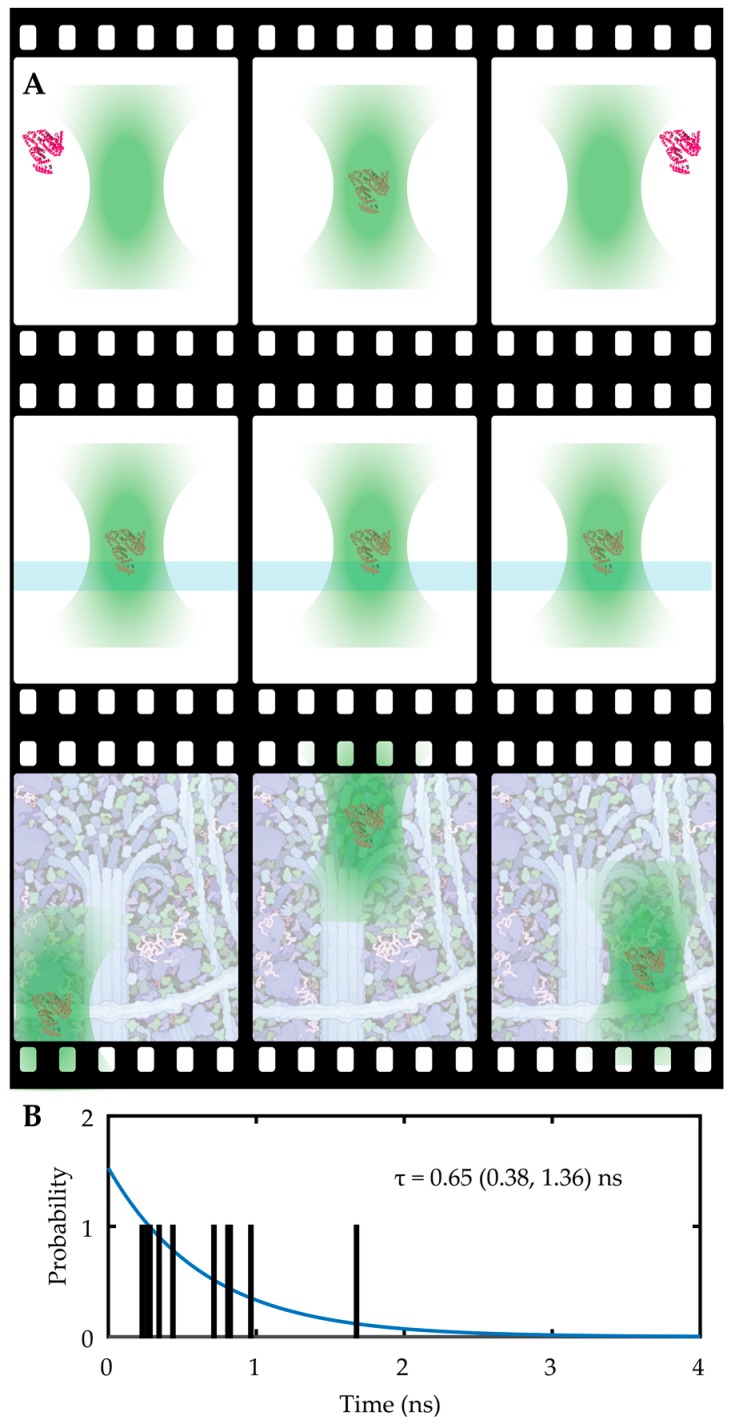
(**A**) Top row: The solution-phase single molecule observation limits measurement time to the diffusion time in the laser focus. Middle row: Tethering to the coverslip extends the observation time but precludes translation to live-cell studies. Bottom row: Real-time 3D single molecule tracking spectroscopy potentially enables continuous observation of freely diffusing proteins in the cellular interior. (**B**) An example of a simulated lifetime of a single fluorophore labelled protein diffusing through a confocal volume, emitting on the order of 10 photons, yielding a large experimental error.

Many biological processes (such as protein folding and molecular diffusion) occur at the microsecond and nanometer scales, requiring new methods for addressing these critical regimes. Single molecule spectroscopy, or the measurement of the properties of single molecules using a spectral readout, offers the possibility to measure molecules with extremely high spatial and temporal resolution. To date, single molecule spectroscopy has been limited to several different methodologies, each with their own tradeoff. The earliest, and in many respects the simplest, implementation of single molecule spectroscopy is confocal solution-phase single molecule spectroscopy (See Figure 2A) [45,46]. In this method, a laser excitation is focused tightly into the sample, creating a diffraction-limited excitation volume. As single molecules diffuse into the focal volume, a sensitive detector (usually a single photon counting avalanche photodiode (SPC-APD)) collects a burst of photons, yielding information on molecular diffusion, fluorophore environment (from lifetime analysis), and even molecular conformation in the case of FRET labelled molecules and a pair of detectors. The benefits of such a method are manifold. First, the ability to use an SPC-APD means the temporal resolution on photon arrival data can be on the order of hundreds of picoseconds, enabling sensitive measurement of fluorescence lifetimes. Second, aside from a fluorescent label, no chemical modification of the molecule is needed. Third, even though only one molecule can be measured at a time, this method can be relatively high throughput given that many molecules can be measured in sequence within a certain experiment. The drawbacks of this method all stem from the limited observation time of the molecule in the focal volume. For example, a 488 nm excitation is expected to have a point-spread function (PSF) with a lateral extent of $\frac{0.61\lambda }{NA}$ and an axial extent of $\frac{2\lambda }{NA^{2}}$, where NA is numerical aperture of the objective and λ is the wavelength of laser. Considering that diffusion in the smaller XY plane will be the limiting factor in determining observation time, the observation area is approximately 200 nm × 200 nm or 0.04 µm^2^. A single protein, with hydrodynamic radius on the order of 3–5 nm, is expected to have a diffusion coefficient in the order of 50 µm^2^/s in water or buffer solution. This means that a single, freely diffusing protein only spends ~1 ms in the focal volume, so the longest timescale process that can be observed is on the order of milliseconds at best. This limited observation critically affects the precision with which certain parameters can be measured. For example, a single fluorophore may only yield 10 kHz collected emission intensity at high laser fluence, meaning that for a 1 ms observation only 10 photons can be collected. This has a dramatic effect on the precision of the parameters extracted from each molecule. For instance, the lifetime measured from a mere 10 photons can be extremely noisy, as shown in shown in Figure 2B. The critical shortcoming here is not just the limited observation time, but also the limited information acquired per molecule.

The most productive single molecule spectroscopy methods have been those which overcome this limited information barrier by tethering or localizing the molecule in some way. Tethered molecules can then be measured with low throughput and high temporal resolution using a confocal detection geometry or with higher throughput and a temporal resolution limited by camera readout in total internal reflection fluorescence microscopy (TIRFM) geometry [47,48]. These two methods have been the workhorses of single molecule spectroscopy. However, both are limited by the need to remove a biomolecule from its native environment and isolate it in a measurement volume or chamber. Several methods have taken confinement in a different direction, where the molecule is still confined in some way but there is no chemical tether. These include using liposomes [49], convex lens-induced confinement (CLIC) [50,51], and the anti-brownian electrokinetic (ABEL) trap [52,53,54,55,56]. While these approaches remove the need for a chemical tether, they still require removing the molecule from its native environment, precluding the observation of single molecule dynamics in complex non-equilibrium environments, such as the cellular interior.

To advance single molecule spectroscopy away from surface bound or tethered methods, a new approach is needed which can maintain a molecule within an observation volume without perturbing the molecule or its environment. A method which endeavors to do just that is real-time 3D single particle tracking (RT-3D-SPT). This review details the different approaches of RT-3D-SPT and gives a perspective on the future applications of RT-3D-SPT to single molecule spectroscopy.

As an introduction to RT-3D-SPT, a differentiation between real-time and image-based single particle tracking methods is firstly required. The key differentiator between real-time and traditional single particle tracking is the emphasis on speed. Traditional single particle tracking (SPT) microscopy allows the observation of the dynamics of a single target particle or molecule using conventional imaging methods [22,57,58]. In traditional SPT, a series of images are acquired. Within this set of images, the particles are identified, and particle locations are concatenated to form trajectories. For these methods, the temporal resolution is determined solely by the imaging rate. For example, when using a TIRF or epifluorescence imaging mode, the imaging rate might be limited to 10 to 200 ms, depending on the readout time of the camera and the required exposure time to overcome read noise. Algorithms such as mean square displacement (MSD) can then be used to analyze the dynamics of the particle. Although two-dimensional single particle tracking has been applied in many studies, the lack of axial information can lead to inaccurate MSDs and biased conclusions [59]. In confocal imaging methods, 3D images are generated by stacking 2D slices together. Further, 3D trajectories are then created by concatenating together the 3D location of particles in consecutive volumes. This leads to a scaling of the measurement time, where the temporal precision is now decreased by a factor proportional to the number of planes sampled. For TIRF or epifluorescence-based imaging, an alternative approach to capture axial motion is to modulate the emission so that the shape of the emission image carries information regarding axial depth within the sample. This has been achieved by simultaneously imaging multiple focal places (bi-plane method, [60]) or using a cylindrical lens to introduce astigmatism [61,62]. In an alternative approach, the Moerner group has pioneered the use of engineered PSFs using phase masks or spatial light modulators at the Fourier plane of the microscope for 3D single particle tracking [63,64,65]. These Fourier plane methods have the advantage of tunable range and precision. While these methods all have the benefit of collapsing the axial position information into a single camera exposure, they are still ultimately limited by camera readout. Furthermore, the tracking range in the axial dimension is relatively small, requiring larger numbers of imaging planes (reducing the temporal resolution) or increasingly complex PSFs to reach larger depths (spreading collected photons oven an increasing number of pixels and degrading the signal to noise ratio).

RT-3D-SPT is a completely different approach. Rather than using post-processing algorithms to extract trajectories from a set of images acquired over a predetermined and rigid volume, RT-3D-SPT aims to use real-time information to lock-on to a moving target using active feedback. By doing so, RT-3D-SPT exchanges a rigid observation volume for a dynamic one which follows a particle of interest. The benefit is that this can be done over very large axial ranges with no sacrifice in temporal resolution. The potential benefit of RT-3D-SPT to single molecule spectroscopy is evident. If the active feedback can be made sensitive and fast enough, it could be used to apply SPC-APD based single molecule detection to capture the dynamics of single molecules in varying environments, such as the cytoplasm of living cells, over long times with high temporal precision.

**Figure 3:**
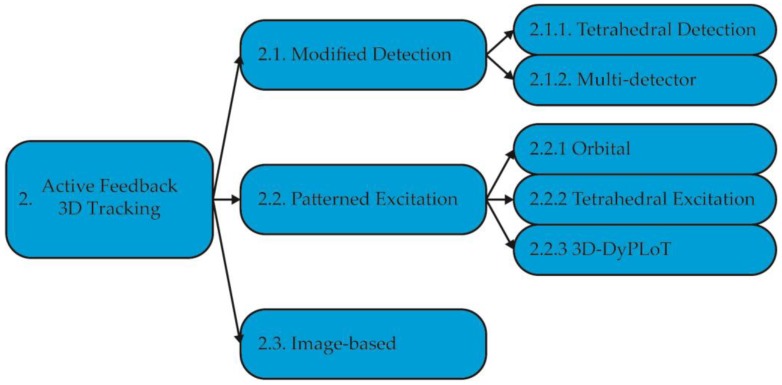
Overview of active feedback tracking methods covered in this review.

This review is separated into 3 parts. First, the different implementations of RT-3D-SPT to date are reviewed (summarized in Figure 3). Next, an often-complicating factor in RT-3D-SPT, the measurement of the contextual environment, is addressed. Finally, the final frontier for RT-3D-SPT—real-time 3D single molecule tracking—is covered.

## 2. Active Feedback 3D Tracking Methods

### 2.1. Active Feedback 3D Single Particle Tracking with Modified Detection

A common implementation of RT-3D-SPT is to use a detection system which can measure particle displacement in real time. From here, they are referred to as modified detection methods. Active feedback tracking with modified detection employs multiple point detectors which correspond to different positions in the object plane. The particle position is estimated in real time by comparing the signal difference between these detectors. This signal difference is then used to drive a piezoelectric stage to move the sample and compensate the particle’s deviation from the center of the detection volume.

#### 2.1.1. Tetrahedral Detection Active Feedback Tracking

**Figure 4:**
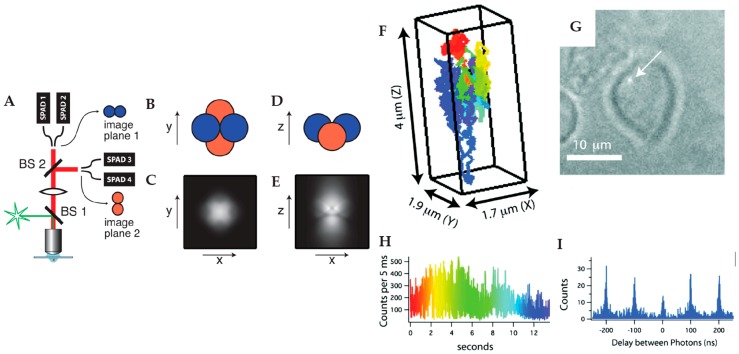
Tetrahedral detection active feedback 3D tracking. (**A**) Schematic of experimental setup. BS: beamsplitter; SPAD: single-photon counting avalanche photodiode. (**B**,**D**) Schematic of the sample volume viewed along Z (**B**) and Y (**D**) axis, in which the four balls represent the sample volumes of four SPC-APDs. (**C**,**E**) Total fluorescent intensity in the Z = 0 plane (**C**) and Y = 0 plane (**E**). Reprinted from reference [67], with the permission of AIP Publishing. (**F**) 3D trajectory of a single quantum dot labeled high-affinity immunoglobin E receptor (IgE-FcεR) in a live cell. The rainbow color from red to blue indicates time. (**G**) Image of cell and the actively tracked receptor (white arrow). (**H**) Fluorescence intensity of the quantum dot probe as a function of time. (**I**) Photon pair correlation measurement shows fluorescence photon antibunching, indicating a single quantum emitter. Reprinted with permission from reference [70]. Copyright 2010 American Chemical Society.

Inspired by feedback tracking with six photomultipliers detecting three-dimensional position developed by Berg et al. [66], Werner and coworkers proposed the first tetrahedral detection tracking method (Figure 4) [67,68]. In this method, four fiber coupled SPC-APDs are arranged to project them onto the object space in a tetrahedral pattern. Two SPC-APDs are used to probe the X position of the target particle while the other two SPC-APD are used to probe the Y position. The SPC-APD pairs for X and Y detection are offset along the optical axis so that the signal difference of these two pairs can be used to calculate the Z position of the tracked particle. Using the signal differences among the various pairs of detectors, the real-time 3D particle position is measured and used to drive a high-speed piezoelectric stage to compensate for the particle’s motion. An early demonstration of this method was able to actively track semiconductor quantum dots in 80% glycerol in a high background environment [69]. This method was extended to active feedback 3D tracking in live cells, as demonstrated by Wells et al., showing the ability to translate into biological systems [70]. By adding another detection channel, Keller et al. further showed the ability to perform multicolor active feedback tracking by tracking multicolor labeled fluorescent beads [71].

#### 2.1.2. Multi-Detector Active Feedback Tracking

**Figure 5:**
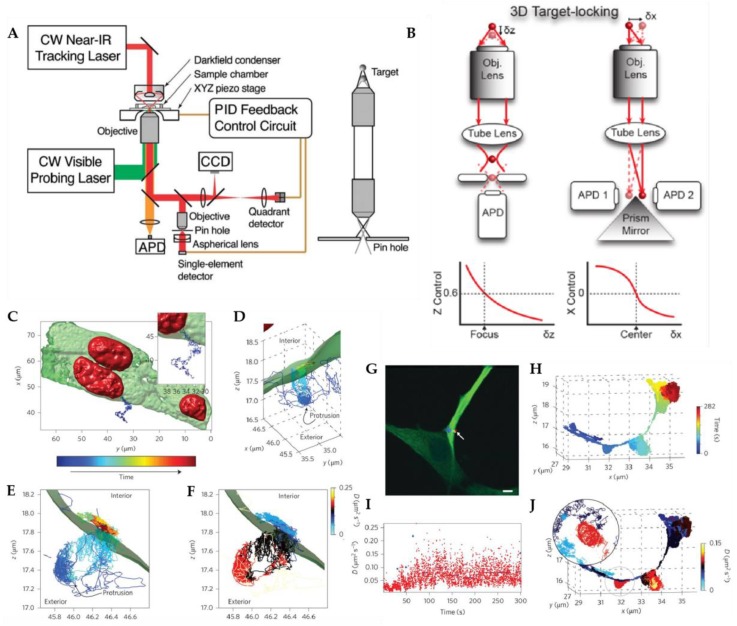
Multi-detector active feedback 3D tracking. (**A**) Schematic of dark-field multi-detector active feedback tracking system developed by Cang et al. which utilized a quadrant photodiode. Reprinted from reference [72], with the permission of AIP Publishing. (**B**) Schematic of multi-detector setup used for fluorescent nanoparticle tracking. (**C**) Top-down view of a polystyrene-quantum dot-peptide nanoparticle (PS-QD-peptide) landing on a membrane protrusion of a live cell. (**D**,**E**) High-resolution view of trajectory in (**C**) with different angles. (**F**) Dynamics heat map of the trajectory, showing regions of varying particle diffusivity depending on its location on the nanoscale protrusion (**C**). (**G**) Overlaid image of two-photon live cell image and high-resolution 2D trajectory. (**H**) High-resolution 3D trajectory of a PS-QD-peptide as it traces out hemispherically-capped protrusions on the live cell membrane. (**I**) Diffusional states as a function of time analyzed by change-point diffusion analysis. (**J**) Dynamics heat map of trajectory in (**H**). Adapted from reference [76] with permission of the author.

At approximately the same time, an alternative modified detection RT-3D-SPT method was developed by Haw Yang and co-workers (Figure 5). The first demonstration was by Cang et al., who implemented a confocal based 3D tracking method with dark field illumination, in which a quadrant avalanche photodiode was used to determine the lateral particle position, while the axial position was determined by the intensity gradient after a confocal pinhole [72]. This method was then modified for fluorescent particle tracking by using four SPC-APDs to replace the quadrant avalanche photodiode [73]. In this system, two prism mirrors were used to split the fluorescent signal onto two pairs of SPC-APDs. The deviation of the particle from the laser focus induced an intensity difference in the paired SPC-APDs which was to calculate the real-time XY position of the particle. This multi-detector active feedback tracking successfully measured the spectral anisotropy of single gold nanoparticles using the correlation between translation and rotational diffusion [74]. Yang et al. further demonstrated the power of active feedback tracking by developing a method which not only held a particle locked in the focal volume, but also actively steered it to a desired location, using the thermal gradient across an illuminated Janus particle [75].

### 2.2. Active Feedback 3D Single Particle Tracking with Patterned Excitation

In the detection-based active feedback tracking methods above, the spatial information was extracted via the arrangement of detectors to collect emission from different areas around a focused laser spot. The alternative methods sought to collect the position information instead with customized excitation patterns. These are referred to as patterned excitation methods. In patterned excitation RT-3D-SPT, instead of using multiple detectors, temporally varying the laser excitation is applied and the particle position is inferred from the temporal response of the collected fluorescence photons, either using a rotating laser beam (Orbital tracking, Section 2.1) or distinct laser foci (TSUNAMI, Section 2.2, and 3D-DyPLoT, Section 2.3)

#### 2.2.1. Orbital 3D Tracking Methods

**Figure 6:**
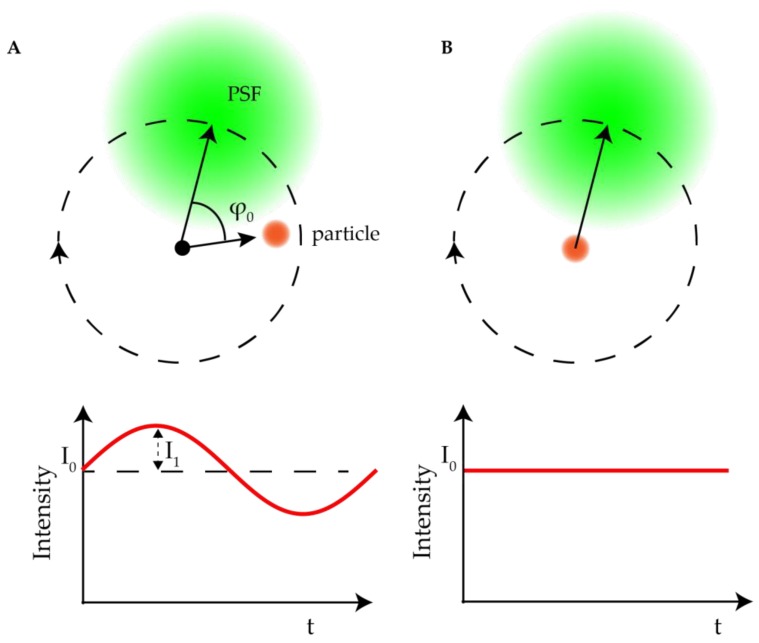
Concept of orbital tracking. The laser (green) is scanned laterally in a circular pattern around a particle (orange dot). (**A**) When a particle deviates from the center of the orbital scan, the fluorescence intensity ($I_{0}$, red curve in the low panel) of the particle is modulated at the same frequency as the laser scan. The phase ($\phi_{0}$) and intensity ($I_{1}$) are extracted via FFT or lock-in amplifier and used to calculate the position of the particle within the orbit. The calculated position is used to relocate the particle so that the modulation of intensity is minimized. (**B**) When a particle is located in the center of the orbital scan, the fluorescence intensity of the particle remains stable with time.

The first modified excitation active 3D tracking method was based on the so-called orbital tracking. The orbital tracking method was first invented by the Enderlein lab for tracking the diffusion of membrane-bound fluorescent molecules [77]. This idea was extended to active feedback 3D tracking by Gratton and coworkers [78] using the ideas based on scanning fluorescence correlation spectroscopy (FCS) [79]. Figure 6 shows an illustration of how the orbital tracking method works. A focused laser beam is driven in a circular pattern in the XY plane using a pair of galvanic mirrors with a diameter on the order of the width of the point spread function (PSF) of the laser. For a particle located precisely at the center of the circular scan, its collected emission intensity is constant in time. If the particle deviates from the center of the circle, its emission intensity fluctuates at the frequency of the laser scan, yielding a time-varying signal proportional to the particle’s position in the XY plane.

Kis-Petikova et al. first used this modulation method to calculate the XY particle coordinates by the fast Fourier transform (FFT) of the intensity signal in 2004 [80]. When the particle is not located in the center of laser scanning circle, the fluorescence intensity I(t) fluctuates sinusoidally with time t: $I\left(t\right)\approx I_{0}+I_{1}cos\left(\omega t-\pi -\phi_{0}\right)$, in which $I_{0}$ is the average fluorescence intensity, $I_{1}$ is the amplitude of fluorescence intensity oscillations and ω is the orbital scanning frequency. The distance between the particle and the center of the orbit can be measured from the amplitude of oscillation $I_{1}$ and the particle’s phase within the circular orbit is determined by the phase $\phi_{0}$. Both $I_{1}$ and $\phi_{0}$ can be easily calculated by FFT in real time. A benefit of this method is the suppression of noise signals at frequencies other than laser scan frequency. To extend this method into 3D tracking, a piezoelectric stage was used to shift the focus between the two axial planes. The Z position of the particle could then be calculated from the difference of the fluorescence intensity in the two focal planes $\frac{I_{00}-I_{01}}{I_{00}+I_{01}}$ ($I_{00}$ and $I_{01}$ are the fluorescence intensities in two focal planes respectively). Using the real-time 3D particle position relative to the orbital laser scan, active feedback is achieved by moving the center of the galvo scan to track the particle’s position. This orbital tracking method is applicable with both 1 and 2 photon excitation, as demonstrated by Levi et al. in 2005 [81] who tracked the phagocytosis of fluorescent beads by fibroblast cells. The temporal resolution in 3D orbital tracking is defined by the orbit frequency, down to 32 ms with a galvo scanner with a concomitant spatial resolution of down to 20 nm (calculated by tracking immobilized fluorescent beads and calculating the standard deviation of XY and Z coordinates). Despite its seeming complexity, Gratton and coworkers demonstrated the ease of implementation of this method by building a 3D orbital tracking microscope onto a commercial confocal laser scanning microscope with minimal modifications in 2014 [82]. In this orbital tracking microscope, instead of rotating the laser beam, the sample was rotated in XY by a piezoelectric nanopositioner. Despite the relatively slow scanning speed of the piezo, the transport of acidic vesicles in live polarized OK cells was easily measured.

In a further improvement to remove the need to move the sample or objective to achieve active feedback 3D tracking, Annibale et al. utilized an electrically tunable lens (ETL) to replace the piezoelectric nanopositioner for axial scanning [83]. The ETL’s focal length is controlled by changing the supplied current, enabling the laser focus to be switched between the two focal planes rapidly without mechanical movement of the objective. The use of the ETL improved the temporal resolution from 32 ms to 8 ms with a demonstrated axial tracking range of up to 300 µm while tracking fluorescently labeled loci in live cells.

**Figure 7:**
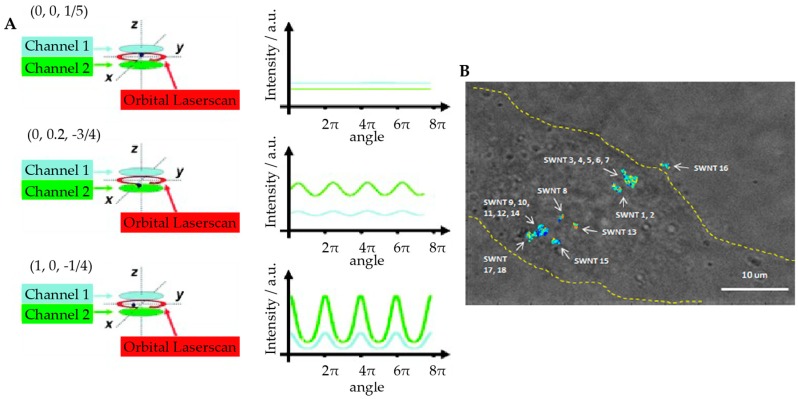
Orbital tracking with biplane detection. (**A**) Schematic of particle position measurement. The laser focus is scanned in a circle around the tracked particle while the two detection channels monitor the intensity at two axial planes (right panel). The X and Y positions of the particle are calculated from the modulation amplitude and phase of the fluorescence signal. The Z position is calculated by the difference of the signal between the two detection channels. Reprinted from reference [84]. Copyright 2009 Wiley-VCH. (**B**) 3D orbital tracking of single-walled carbon nanotubes (SWNT) in live cells. The image shows overlaid bright field image of Hela cell (outlined in yellow) with 18 SWNT trajectories. Adapted from reference [85]. Copyright 2012 American Chemical Society.

Further, realizing that the slow step in previous 3D orbital tracking implementations was determined by the axial scan speed, Lamb and co-workers replaced the axial scan with two SPC-APDs to monitor the two offset focal planes [84]. This method was realized by splitting the fluorescent signal with a 50:50 beamsplitter and using two confocal pinholes corresponding to two different Z planes placed before each SPC-APD. The axial position of the particle is then obtained by the relative fluorescence intensities between these two detectors. F. Reuel et al. applied this method to study the trafficking of single-walled carbon nanotubes (SWNT, Figure 7) [85], showing that the translational and rotational characteristics of SWNTs could be used to measure the microscopic viscosity in live Hela cells. As an added benefit, the SWNTs demonstrated a marked increase in photostability compared to fluorescent beads and consequently much longer trajectory durations were achieved. The combination of orbital tracking and SWNTs, which demonstrate a large Stokes shift and NIR emission [86,87], indicates this method might be optimal for active feedback tracking deep in the tissue or other turbid environments.

**Figure 8:**
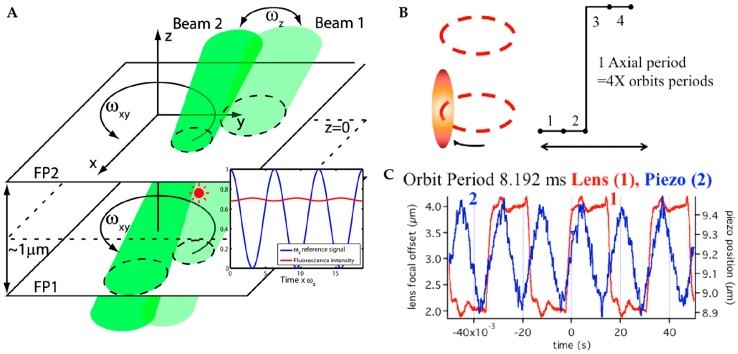
(**A**) Schematic of orbital 3D tracking with high-speed laser modulation using acousto-optics. Two laser beams with rotation frequency $\omega_{xy}$ are focused at two different depths in the sample separated by ~1 µm. The excitation power is alternated between these two laser beams with a frequency of $\omega_{z}$. The magnitude of the $\omega_{z}$ frequency component in the fluorescence intensity is proportional to the particle’s distance from z = 0. Adapted from reference [88]. Copyright 2007 American Chemical Society. (**B**) Schematic of 3D orbital tracking using an ETL. A complete 3D tracking period is comprised of two orbits above the particle and two orbits below the particle. (**C**) Comparison of the response time of piezo stage versus ETL for an orbital scanning period of 8.192 ms, with the ETL showing a much faster response. Adapted from reference [83]. Copyright 2015 Optical Society of America.

The above methods share the limitation that the ultimate temporal resolution is limited by the scan frequency of the orbit. Mabuchi and coworkers pushed the limits of the orbital tracking method using two high frequency modulated ($\omega_{z}$) excitation beams in the two Z planes with a separation of 1 µm to illuminate a fluorescent particle [88] (Figure 8). In this method, acousto-optic modulators (AOM) were adopted to rotate the laser beam instead of the galvanic mirrors. Similar to the lateral position calculation in orbital tracking, the particle’s Z position was now also encoded in the fluorescence intensity signal by switching rapidly between to the focal planes. The oscillation magnitude of fluorescence intensity in the $\omega_{z}$ frequency component is proportional to the distance of the particle from the origin while the phase shows the direction (above or below the focal plane). With this method, a single quantum dot was tracked in water with D ~20 µm^2^/s at count rates near 100 kHz, which remains the fastest reported 3D active feedback tracking to date. Berglund and coworkers further modified the 3D orbital tracking microscopy by replacing the AOMs with acousto-optic deflectors (AOD) [89]. This tracking method was used to investigate the behavior of nanoparticles at the silicone oil−water interface and found there was a non-linear relationship between the diffusion coefficient and the particle size for small particles in the surface. The binding of DNA origami and quantum dots was also investigated with this method [90].

#### 2.2.2. Tetrahedral Excitation 3D Tracking

A complementary method to the tetrahedral detection method is the tetrahedral excitation method. Instead of using a set of four SPC-APDs, tetrahedral excitation tracking utilizes four alternating laser pulses for fluorescence excitation. The four laser foci are placed in a tetrahedral pattern, with each spot occurring at a well-defined time. The fluorescence is collected by a single SPC-APD and the position of the tracked particle is calculated from the photon differences detected from each excitation spot. The calculated position is then used to drive a piezo stage to reposition the particle to the center of tetrahedra excitation volume.

Davis and coworkers developed the first tetrahedral excitation-based tracking microscopy by using four alternately pulsed laser diodes [91]. The laser diodes were combined using beamsplitters and overlapped in a tetrahedral geometry at the focus of the objective lens. A high-speed counter/timer card was used to modulate the four laser diodes and count photon arrival pulses from the SPC-APD. Once the particle’s position was estimated by comparing the photon counts in each axis, a feedback loop was applied to drive the piezoelectric nanopositioner to compensate for the particle’s position deviation from the center of the four laser foci with a temporal resolution of 1.86 ms, based on the time to cycle between the four laser pulses. The particles with diffusivity up to 12 um^2^/s were tracked with this tetrahedra excitation tracking method although the tracking duration was only ~100 ms.

**Figure 9:**
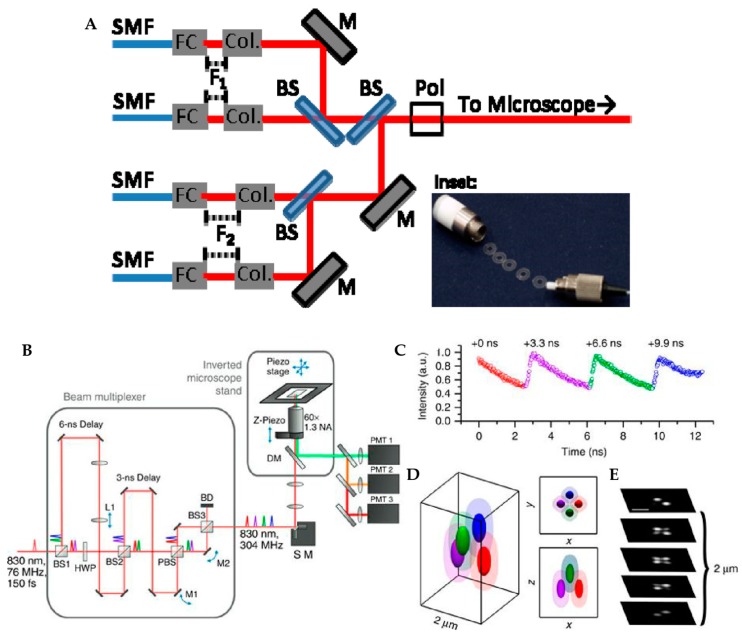
(**A**) Schematic of tetrahedral excitation tracking setup, where four laser diode beams are combined by three beamsplitters and focused onto the sample in a tetrahedral pattern. (inset) Washers inserted between the fiber coupler (FC) and collimator (Col) are used to arrange the four laser foci after the objective. Adapted from reference [91]. Copyright 2015 Optical Society of America. (**B**) Schematic of TSUNAMI. A pulsed laser is split by two beamsplitters to generate four laser beams. The temporal separation between four beams is achieved by physical delay. The arrangement of the four foci after the objective is controlled by mirrors in the excitation beam path. (**c**) Temporal separation of the four excitation beams measured by the fluorescence of an emitter in the center of the detection volume. (**D**) Schematic of the tetrahedral PSF. (**E**) Scanning mage of 100 nm fluorescent beads in different depth. Scale bar: 2 μm. Adapted from reference [92] under Creative Commons Attribution 4.0 International License.

In a further improvement of this method, Perillo et al. replaced the four laser diodes with a mode-locked Ti:Al_2_O_3_ pulsed laser split into four separate foci. A temporal delay between these pulses was created by physical delay lines with 3.3 ns delay between each spot (Figure 9) [92]. After passing through the objective lens, these four excitation pulses formed a tetrahedral shaped excitation volume in the sample space. This tetrahedra excitation tracking method, called tracking single particles using nonlinear and multiplexed illumination or TSUNAMI, was implemented with a two-photon excitation. The spatial localization precision of TSUNAMI can reach 35 nm (demonstrated on a 100 nm fluorescent bead with 2 µm/s velocity). The temporal resolution of TSUNAMI was demonstrated to be down to 50 µs although the system response time was 1 ms, as limited by the piezoelectric stage.

#### 2.2.3. Dynamic Photon Localization Tracking (3D-DyPLoT)

**Figure 10:**
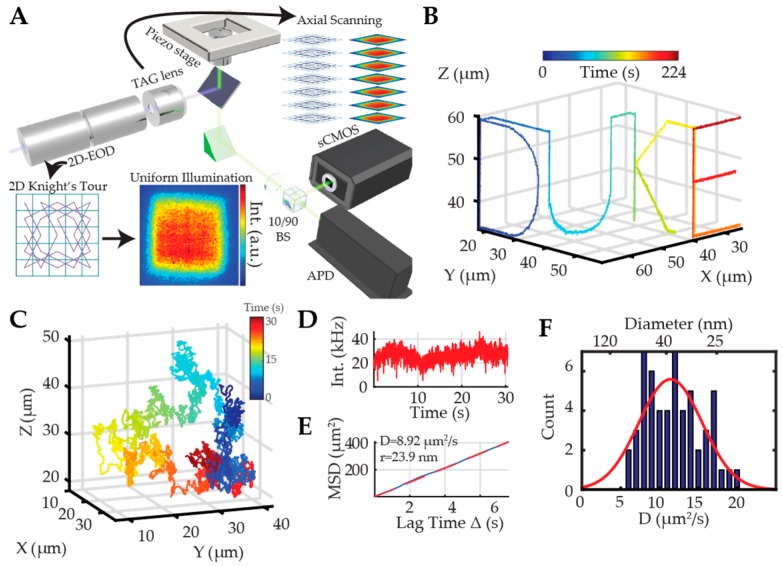
(**A**) Schematic of 3D-DyPLoT. A 2D-EOD is used to deflect the laser in X and Y in a knight’s tour pattern (20 μs per spot) while a TAG lens dynamically modulates the focus at ~ 70 kHz. (**B**) Fixed 190 nm fluorescent particle is driven along a predetermined pattern by a micro-stage and tracked by 3D-DyPLoT. (**C**–**F**) 3D-DyPLoT is used to track silica-coated giant nonblinking quantum dots (gQD) in water. (**C**) 3D trajectory. (**D**) Fluorescence intensity of gQD as a function of time. (**E**) MSD analysis of a free diffusing gQD. (**F**) Histogram of diffusion coefficient and calculated diameter of gQD showing a mean hydrodynamic diameter of 38 nm. (inset) TEM of silica-coated gQD. Adapted from reference [93]. Copyright 2017 Optical Society of America.

Recently, the Welsher lab developed a robust real-time active 3D tracking method called 3D dynamic photon localization tracking (3D-DyPLoT) [93,94]. In this method, a two-dimensional electro-optic deflector (2D-EOD) and a tunable acoustic gradient (TAG) lens were used to dynamically deflect the laser focus in three dimensions at a high rate (50 kHz XY, 70 kHz Z, Figure 10A). The arrival times of collected fluorescence photons were used to estimate the particle’s position by correlating the photon arrival time with the laser focus position using an assumed Gaussian density Kalman update filter developed by Fields and Cohen [95]. The lateral scanning was realized by the 2D-EOD, which drives the laser focus in a 1 × 1 µm knight’s tour pattern, inspired by the ABEL trap [52]. The axial scanning was achieved with the TAG lens, which can change the divergence of the laser beam dynamically to create a 2–4 µm scanning range in the imaging plane [96,97,98]. After calculation of the particle position with the optimized position estimation algorithm in 3D, the feedback control is applied to the piezoelectric nanopositioner to follow the particle’s motion (Figure 10B). The main differentiator between 3D-DyPLoT and tetrahedral excitation or orbital tracking is the large scanning area. This large scanning area allows the tracking system to recover from large diffusive jumps, emitter dark periods, and lag of the piezoelectric actuators. This high speed and large scan area enables 3D-DyPLoT to track rapidly diffusing particles (up to 20 µm^2^/s) at low photon count rates (20 kHz, Figure 10C–F). This method has also been demonstrated to work well with biologically relevant samples, allowing the tracking of single lentiviral particles fused with yellow fluorescent proteins (YFP) over long periods of time. Apart from the high tracking robustness at low photon count rates, 3D-DyPLoT is able to achieve sub-10 nm lateral and 15 nm axial (demonstrated by tracking an immobilized fluorescent bead). The temporal resolution can be up to 20 µs at high signal rates and is otherwise simply limited by the count rate. This method is also extendable to single cell tracking in living organisms. By using galvanometric mirrors to replace EODs for XY scanning, Karagyozov et al. built a similar two-photon 3D active tracking system and used it to investigate the calcium dynamics of neurons in unrestrained freely behaving fruit fly larvae [99]. These results show active feedback 3D tracking can be a powerful tool for neuroscience research and can be extended to the small organism level.

### 2.3. Image-Based Active Feedback Tracking

**Figure 11:**
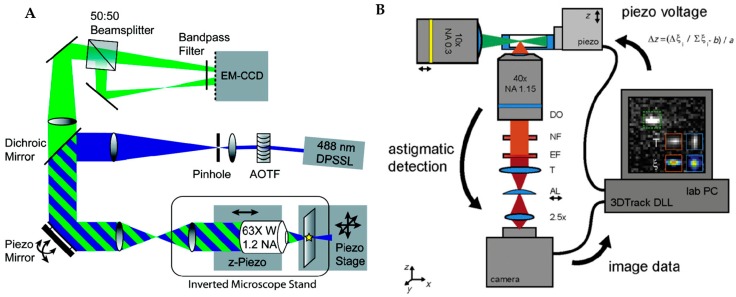
(**A**) Schematic of image-based biplane active feedback 3D tracking. One part of fluorescence is transmitted through a 50:50 beamsplitter and imaged onto the EMCCD camera while the other part of fluorescence is reflected by beamsplitter and imaged on a different side of the EMCCD. The reflected fluorescence has a longer path so that two axial planes can be monitored synchronously. The piezo mirror and Z piezo stage are used to move the laser focus in 3D. Adapted from reference [100]. Copyright 2010 American Chemical Society. (**B**) Schematic of astigmatic imaging active feedback 3D tracking. A high NA objective is used for light sheet illumination and the 3D information of the particle is encoded in the astigmatic image. The image is analyzed and a feedback control is applied to the Z piezo stage to maintain the particle in focus. Adapted from reference [101] under Creative Commons CC BY Licence.

The image-based active feedback tracking methods using EMCCD or sCMOS cameras as detectors have also been demonstrated. While these methods lack the temporal resolution and signal-to-noise of SPC-APD methods, they do have the added benefit of built in contextualization. Bewersdorf and Juette developed such a feedback tracking method by combining bi-plane imaging with active feedback tracking [100]. In this method, the fluorescence from a particle is detected in two detection planes on separate areas of an EMCCD. The relative images of these two focal planes are used to determine the axial position of the particle. A piezoelectric mirror and a piezoelectric-actuated objective holder are used to relocate the particle to the focal plane with a real-time feedback mechanism (Figure 11A). While the use of the EMCCD maximized the detection efficiency compared with confocal-based tracking methods, it should be noted that this method is not truly real-time because the temporal resolution is still limited by the readout time of cameral (3.2 kHz in the current method). Another image-based active feedback tracking method was developed by Spille et al., who combined light sheet illumination with active feedback tracking [101]. In this method, an astigmatic cylindrical lens was used to create an asymmetric PSF along the axial direction. The 3D position information was encoded in the shape of the image of the particle and a Z piezoelectric stage was used to keep the particle in the focal plane (Figure 11B). Due to the light sheet illumination, this tracking method demonstrated a lower background compared with the bi-plane method. The exceptionally high signal-to-noise ratio (SNR) allowed 3D localization of a single molecule with just 100–400 photons in one frame. This light sheet based active tracking method was used to investigate mobility transitions of ribosomal (r)RNA and messenger (m)RNA particles in cell nuclei.

## 3. Method Comparison

To facilite the selection of a real-time 3D tracking method for a particular application, the localization precision, temporal resolution, fastest tracked diffusive dynamics and reported tracking length at the fastest tracked dynamics of these active feedback tracking methods are listed below (Table 1). Clearly, there are pros and cons to each method. Typically, the trade-off is between the localization precision, temporal resolution, tracking speed (diffusive) and tracking duration. Even for one particular tracking method, the appropriate feedback parameters based on the experimental requirements also need to be chosen. For example, more aggressive feedback can lead to tracking more rapidly diffusing particles for longer times, but at the expense of localization precision.

## 4. Environmental Contextualization in Active Feedback-Based 3D-SPT Methods

As demonstrated above, 3D-SPT methods featuring active feedback mechanisms offer unprecedented temporal resolution which enable measurement and quantification of dynamic cellular processes. However, the trajectories obtained by these methods cannot measure the interactions between the tracked particle and the greater cellular environment which drive many important biological questions. Several groups have sought to contextualize trajectories by incorporating the ability to image the region surrounding the probe.

**Figure 12:**
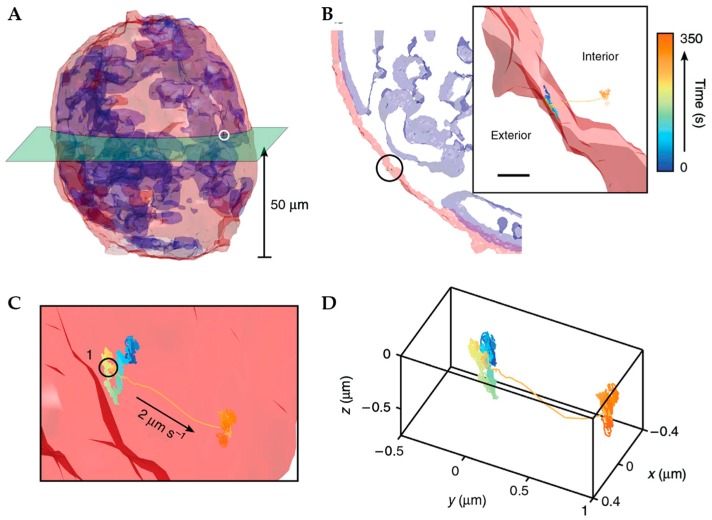
Prior volumetric imaging combined with TSUNAMI-based tracking was used to acquire a volumetric image of the entire 100 µm diameter tumor spheroid around an internalizing particle. (**A**) 3D isocontour reconstruction of tumor spheroid with red plasma membrane and blue nucleus. Green section at 50 µm shows plane of EGFR internalization trajectory. (**B**) Isocontour model of the green slice in (**A**). Inset shows zoomed in view highlighting spheroid boundary and the placement of the trajectory as it transports into the spheroid. (**C**) Zoom in (**B**). (**D**) Isolated trajectory. Adapted from reference [92] under Creative Commons Attribution 4.0 International License.

TSUNAMI contextualizes its trajectories using a two-photon image acquired prior to RT-3D-SPT [92]. Multiple images are scanned sequentially at different focal depths to generate full volumetric data which are spatially correlated to the later obtained trajectories. The method captured volumetric imaging data of A431 tumor spheroids with a diameter of 100 µm and traced epidermal growth factor receptor (EGFR) internalization pathways at multiple depths within the spheroid. As the field of view for imaging was ~100 µm × 100 µm, they were able to image the entire spheroid and then visualize the trajectory region deep within due to the multiphoton tracking excitation (Figure 12). While this approach to contextualization allows for the acquisition of volumetric images of the entire cell, it eliminates the possibility of capturing any broader cellular changes due to the temporal difference between tracking and imaging timepoints. It should also be noted that while this was demonstrated with the TSUNAMI tracking method, the idea of prior acquisition of cellular images is applicable to all of the active feedback methods described above.

**Figure 13:**
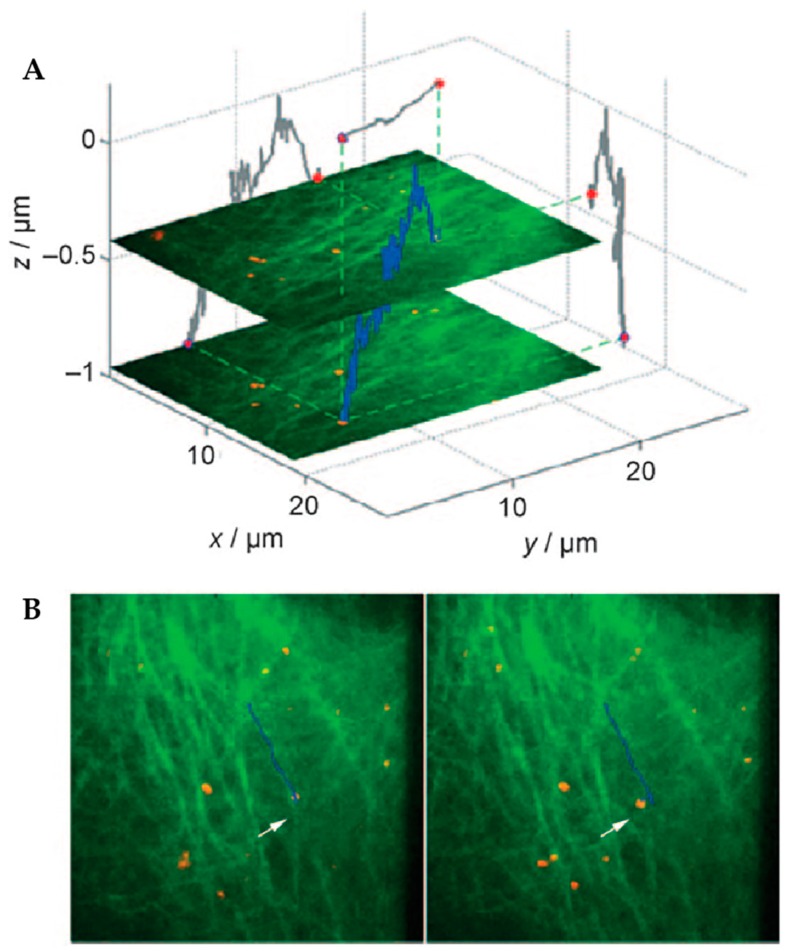
The addition of simultaneous widefield imaging to orbital tracking allowed trajectories (blue) of artificial viruses (red) to be placed in the environmental context of their interactions with the cytoskeleton as visualized with eGFP-labeled tubulin (green). (**A**) Trajectory of an artificial virus shown in 3D space with 2D projections across each axis shown in gray. (**B**) Two frames in a time series of images with trajectory overlaid. In the left frame, the particle is moving laterally not because the virus itself is changing microtubules, but because the microtubule itself was moving. This observation could not have been confirmed without simultaneously acquired imaging. Reprinted from reference [84]. Copyright 2009 Wiley-VCH.

Other methods have contextualized the surrounding environment by performing simultaneous imaging of the region surrounding the probe through secondary optical setups which are locked to the focus of the moving particle. Katayama et al. [84] added context to orbital tracking through simultaneous widefield imaging. This imaging was performed by separating the tracking and imaging emission using a dichroic mirror and using a second dichroic mirror to spectrally separate the imaging emission onto different regions of an EMCCD camera. As the objective is mounted on a Z piezoelectric nanopositioner which moves with the tracked feedback, the image focus is probe-locked. Using this method, they visualized the motion of an artificial virus along the enhanced green fluorescent protein (eGFP)-labeled Tubulin in HUH7 cells across an image size of ~42 µm × 21 µm with pixels sampled every 82.5 nm (Figure 13). The overlay between tracking and imaging channels allowed them to verify that the tracked particle was moving along microtubules and that the stalled motion in their trajectory was the result of a microtubule network rearrangement visible only in the widefield image. Unlike other methods outlined here, the contextual imaging with orbital tracking is similar in spatiotemporal resolution. Both tracking and imaging occur on the millisecond timescale (32 ms tracking temporal resolution and ~200 ms imaging exposure time) and feature the same spatial sampling (pixel size), though the lack of optical sectioning means 3D image information is not collected.

**Figure 14:**
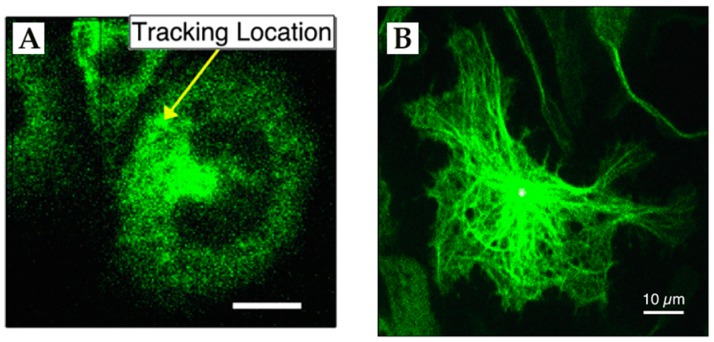
Tetrahedral detection tracking coupled with simultaneously acquired spinning disk confocal images. (**A**) Localization of QD-labeled probe within the cellular structured using LED illumination requiring 300 ms exposure time. (**B**) Laser excitation reduced needed exposure time to 40 ms and provides a much higher contrast image than (**A**). Reprinted from reference [102], with the permission of AIP Publishing.

A method by DeVore et al. [102] contextualized trajectories with simultaneous spinning disk confocal imaging. The benefit of confocal imaging provided optical sectioning localized to the focal plane of the tracked particle. They initially acquired images with a 300 ms exposure time when utilizing LED excitation. While this led to the successful tracking of QD-labeled IgE-FcεRI inside rat mast cells with YFP-labeled tubulin (Figure 14), the images suffered from stage-motion induced blurring due to the long exposure time and parallel nature of the acquisition. Further, changing to laser excitation allowed a reduction exposure time to 40 ms which prevented the blurring and provided higher-contrast sections.

**Figure 15:**
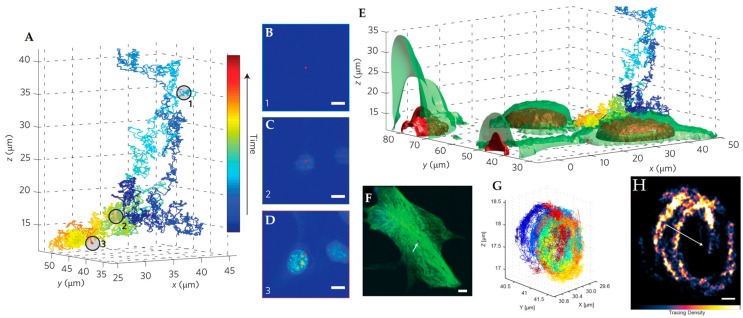
3D Multi-Resolution Microscopy combines RT-3D-SPT with simultaneously acquired 2P-LSM images. (**A**) Isolated trajectory of QD-labeled nanoparticle probe. (**B**–**D**) Corresponding 2P-LSM sections acquired at depths indicated in circled portions of trajectory in (**A**). (**E**) Overlay of co-registered trajectory with 3D reconstruction of interpolated 2P-LSM sections gives cellular context to trajectory motion. Nuclear regions are highlighted in red and cell membrane surfaces are green. (**F**) 2P-LSM maximum intensity projection of internalized QD-labeled probe in a NIH-3T3 fibroblast cell. Arrow indicates position of tracked particle. (**G**) Ellipsoidal trajectory of macropinosomal membrane-bound probe showing multiple curved surfaces due to macropinosomal motion. (**H**) Structural tracing of the trajectory shown in (**E**). Arrow shows center of mass motion during trajectory. Adapted from reference [76] with permission of the author.

Welsher and Yang developed 3D multi-resolution microscopy (3D-MM) which achieved context by utilizing the two-photon laser-scanning microscopy (2P-LSM, Figure 15) [76]. With the real-time tracking the focal plane of the 2P-LSM was locked to the probe and the X and Y coordinates of voxels could be extracted from the piezoelectric stage positions. While the time to draw an entire frame was ~1 s, the individual pixel dwell times were significantly lower than the feedback rate of 10 µs. This prevented the stage-induced motion blurring seen above with parallelized acquisition. This method was applied to contextualize several phenomena, including the binding of extracellular particles to the cell surface, the diffusion of particles on membrane protrusions and filopodia, and the confined diffusion of particles on the macropinosomal membrane. The image field of 64 µm × 64 µm was utilized to place these trajectories into the proper cellular context.

The methods outlined above which achieve simultaneous contextualization do so within the confines of the focus being locked to the probe (summarized in Table 2). Particularly in the methods which produce optically sectioned images, being locked to the focal plane of the tracked particle prevents acquisition of volumetric images. Future efforts to contextualize 3D-SPT methods would be improved by the extension to 3D contextualization. An ideal method would combine the unique strengths of the various methods, while eliminating the weaknesses. The ideal method would utilize only a single tracking detector, have high temporal resolution and spatial precision, and feature probe-decoupled, simultaneous volumetric imaging.

## 5. Active Feedback 3D Single Molecule Tracking: The Final Frontier

**Figure 16:**
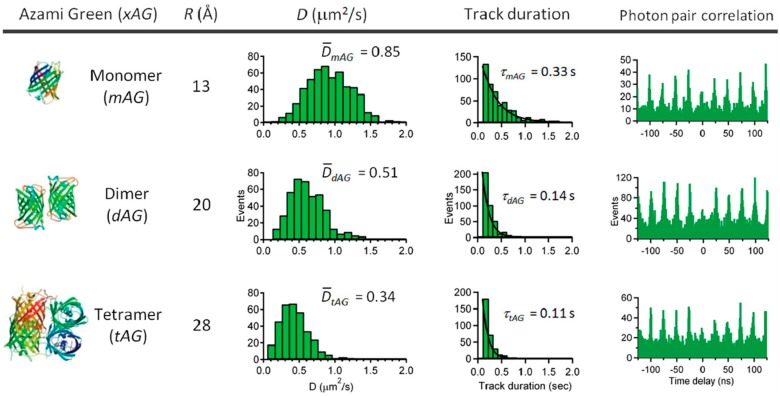
RT-3D-SMT using the tetrahedral detection method reveals the protein oligomerization states of Azami Green oligomers mAG, dAG, and tAG in 92% glycerol solution. Reprinted with permission from reference [104]. Copyright 2012 American Chemical Society.

As detailed above, active 3D single particle tracking methods enable high temporal resolution probing of rapidly moving particles. In multiple cases, these methods were able to stretch the application towards the ultimate goal of tracking a single molecule. Here, the focus is on the tracking of molecules with a single fluorophore, though the earliest single molecule active feedback tracking was demonstrated on multiply-labelled lambda DNA by McHale et al. [103]. The first demonstration of single fluorophore active feedback tracking came from Wells et al., who applied tetrahedral detection tracking to track single dUTP-Cy5 (red cyanine dye; ex: 649 nm; em: 666 nm) molecules in 90% glycerol [70]. While the diffusion coefficient of these single molecules was measured to be approximately 3 µm^2^/s, well within the speed limit of the tetrahedral method (corresponding to a > 100 nm diameter sphere in water), single molecule trajectories did not exceed 0.3 s. Even within these limits, Han et al. extended this approach to track the oligimerization of azami green [104], this time in 92% glycerol. Again, despite the reduction in the diffusive speed of the molecules, trajectory durations were still quite short, remaining on the order of several hundred milliseconds (Figure 16).

**Figure 17:**
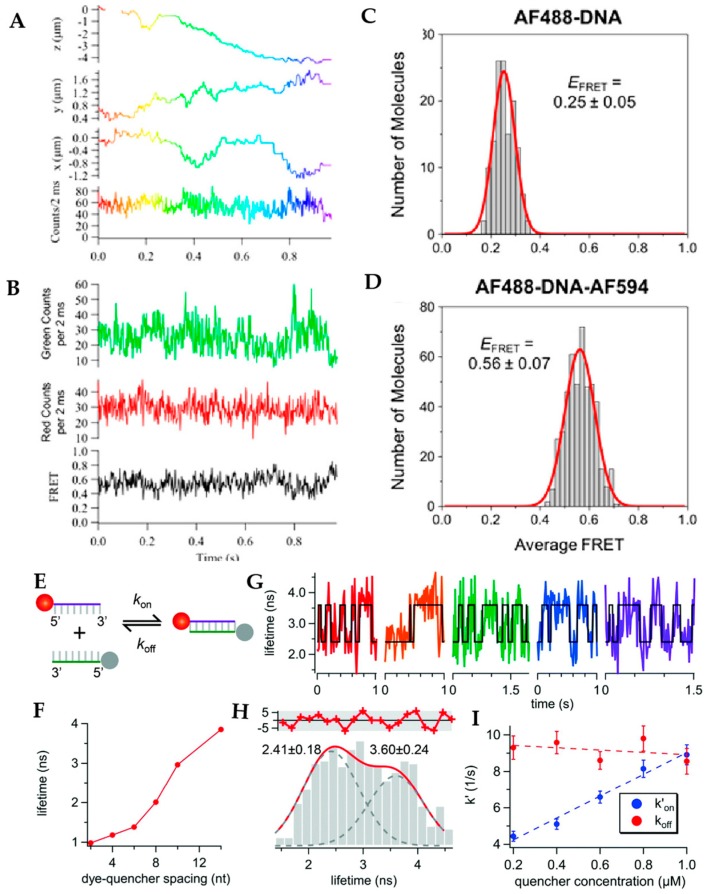
Tetrahedral detection tracking of intramolecular FRET within double-stranded DNA in 90% glycerol-water solution. (**A**) X, Y, Z position and fluorescent intensity of AF488 (Alexa Fluor 488; ex: 490 nm; em: 525 nm)-DNA-AF594 (Alexa Fluor 4594; ex: 590 nm; em: 617 nm) molecule. (**B**) Fluorescence intensity and FRET efficiency as a function of time for the trajectory shown in (**A**). (**C**) Distribution of FRET efficiency of AF488-DNA molecules (control sample). (**D**) Distribution of FRET efficiency of AF488-DNA-AF594 molecules. Reprinted with permission from reference [71]. Copyright 2018 American Chemical Society. (**E–I**) Measurement of the hybridization kinetics of freely diffusing ssDNA molecules in solution by tetrahedral detection 3D tracking. (**E**) Scheme of the donor-quencher system for the DNA hybridization kinetics measurement. Red ball denotes ATTO633 (ex: 630 nm; em: 651 nm) reporter and grey ball denotes Iowa Black quencher. (**F**) Fluorescence lifetimes of ATTO633 with different donor-quencher separation distances. (**G**) Lifetime measurement in 3D tracking of ssDNA in 70 wt% glycerol solution. The fluorescence lifetime switching indicates transient annealing and melting events. (**H**) Lifetime histogram built from (**G**) showing two states. (**I**) Apparent annealing rate (k_on_) and melting rate (k_off_) as a function of quencher concentration shows the annealing rate is quencher concentration dependent. Adapted from reference [105]. Copyright 2017 the Royal Society of Chemistry.

This same method was further applied to track bimolecular interactions in solution at the single molecule level. Keller et al. demonstrated intramolecular single-molecule FRET of double stranded DNA in viscous glycerol solution, showing the capability for single-molecule FRET observation RT-3D-SPT [71]. By combining tetrahedral detection tracking with time-domain fluorescence lifetime measurement, Liu et al. measured hybridization kinetics of free diffusing single stranded DNA (ssDNA) molecules in solution [105]. In this experiment, a fluorescent donor and fluorescent quencher were labeled on two complementary ssDNAs separately and the hybridization of these two ssDNAs were measured by the decrease of the donor’s lifetime due to Förster resonance energy transfer. Compared with hybridization kinetics measurement in 2D, where the molecule is tethered to a coverslip, the 3D measurement should provide a less perturbative environment (Figure 17).

## 6. Outlook

The above studies provide evidence that real-time 3D single molecule tracking spectroscopy is possible. However, there are numerous shortcomings that need to be addressed to reach the ultimate goal of tracking single molecules in aqueous solution. Of the methods covered in this review, only the tetrahedral detection method has demonstrated the ability to track a single molecule. It is not clear why this particular method would be advantageous over all the other methods, but here we can speculate on the possible reasons. The first is that this is a photon counting method. The reason this is important can be seen by examining the high-speed orbital tracking methods above. Mabuchi and coworkers demonstrated that by rotating the laser at a high frequency (~20 kHz), a time-varying intensity can be encoded which represents the particle’s position [88]. According to Nyquist, the lowest count rate that could carry such a signal would be 40 kHz, a challenging though not impossible number to hit for single molecules. Such high intensity would, however, require high excitation powers leading to rapid photobleaching and limiting trajectories. The photon counting methods do not suffer from this issue, using each photon to update the position estimate. This may explain one reason that the tetrahedral detection method has been the only successful method for real-time 3D single molecule tracking thus far.

Digging more into the numbers of the relative successes in real-time 3D single molecule tracking spectroscopy, another pattern emerges. Each successful single molecule demonstration required the use of a highly viscous solution (>90% glycerol) to achieve single molecule trajectories, bringing the diffusion coefficient of the molecules down to < 3 μm^2^/s. This is critical because this is the diffusive speed that corresponds to a sphere of > 100 nm in water, which, in the form of a fluorescent nanoparticle, is easily tracked by most of the methods above. This indicates that the speed of the response should not be limiting. At the outset of transforming a real-time 3D single particle tracking microscope into a real-time 3D single molecule tracking microscope, one might consider two possible roadblocks. First, molecules diffuse very quickly and the piezoelectric or galvanic actuators may not be fast enough to respond. Second, molecules yield very few photons per feedback loop cycle as compared to quantum dots or fluorescent beads. Switching to a high percent glycerol solution would seem to address the first issue. So why are the single molecule trajectories so short (several hundred ms) when the speed issue seemed to be addressed? The answer must lie in the low photon count rates.

Here we propose a reason the low photon counts rates preclude RT-3D-SMT for long periods of time. Looking back at the example from Fig. 2b, we see that a limited number of photons can lead to large uncertainty in experimental parameters. If we consider that the piezoelectric response time (the most common actuator used in RT-3D-SPT) is on the order of 1 ms, one can reasonably expect to detect on the order of 10 photons to calculate a position estimate before the stage can counteract the molecule’s motion. This leads to large uncertainties which may send the stage in the wrong direction. When the stage fails to return the molecule to the center of the observation volume, the photon rate drops as the particle eludes detection and is lost, ending the trajectory. We propose that this is the main determinant of trajectory length in RT-3D-SMT thus far. So what is the solution? The ABEL trap provides a very important example. The ABEL trap, while effectively working in 2D and relying on electric fields to trap a single molecule, operates on the same principle as RT-3D-SPT. It measures the molecule position and applies feedback. The major difference is that the feedback is much faster, on the order of microseconds to change the field. Implementations of the ABEL trap use laser scan patterns which are 2-3 microns wide [52,53,54,55,56], allowing the molecule to be loosely held. Even if the feedback cannot hold the molecule precisely in the center of the trap, the molecule will still provide photons and thus position information as it jumps towards the edges. This is what is needed to push RT-3D-SPT to RT-3D-SMT: acknowledgement that the piezoelectric response is lagging and inaccurate at high speeds and low count rates. As such, the excitation and detection areas of the method need to be increased to compensate. Giving a cushion for the molecule to jump away from the center and allowing the system to recover without losing photons should increase the trajectory length and lead to more fruitful application of this promising technique. Lastly, this increased excitation and detection range is not a cure-all. The limiting factor will still remain the speed of the piezo or galvo actuator response. Future implementations would benefit from implementing alternative feedback actuators, such as high-speed deformable mirrors or spatial light modulators, which have bandwidths far exceeding mechanical actuation [106].

Here we have reviewed the various methods of RT-3D-SPT to date, including attempts to translate to RT-3D-SMT. While much progress has been made, improvements are still needed in terms of sensitive single molecule detection and response speed. If those improvements can be made, RT-3D-SMT promises to be a powerful technique for placing high temporal resolution single molecule dynamics back into their native cellular context.

## Figures and Tables

**Table 1:** Comparison of active feedback RT-3D-SPT methods. (a) Localization precision measurement for each method. (b) Sample and conditions used for the localization precision measurement in (a). QD: quantum dot; FNP: fluorescent nanoparticle; Rate: emission intensity; D: diffusion coefficient. (c) Reported temporal resolution where available. Int.: Integration time; Piezo/Galvo/ETL: respective response times of feedback mechanisms. (d) Fastest succesful tracking of Brownian motion reported. D: diffusion coefficient. (e) Count rate at highest reported diffusion coefficient. (f) Duration of trajectory at highest reported diffusion coefficient. (g) References.

Name	(a) Localization Precision X/Y/Z (nm)	(b) Precision Measurement Sample	(c) Temporal Resolution	(d) Fastest Tracked D (μm^2^/s)	(e) Count Rate (Fastest D, kHz)	(f) Tracking Duration (Fastest D)	(g) References
Tetrahedral detection	70/70/110	Sample: QD	Int.: 5 ms Piezo: ~1 ms	3	~16	0.3 s	[69,70]
		Rate: 40 kHz					
		D: 0.37 µm2/s					
Multi-detector	9.8/12.8/13.1	Sample: FNP	Int.: 10 μs Piezo: ~1 ms	4.81	-	>8 s	[76]
		Rate: -					
		D: Stationary					
Orbital (piezo stage)	2.4/2.8/2.4	Sample: FNP	Piezo: 32 ms ETL: 8 ms	0.04	-	-	[81,82,83]
		Rate: 28 kHz					
		D: Stationary					
Orbital (laser modulation)	352/352/272	Sample: QD	Piezo: 1 ms	20	~120	20 s	[88]
		Rate: ~120 kHz					
		D: 20 µm2/s					
Orbital (bi-plane detection)	15/15/20	Sample: FNP	Galvo: 32 ms	2	50	~25 s	[84,90]
		Rate: 10–40 kHz					
		D: Stationary					
Tetrahedral excitation	60/60/300	Sample: FNP	Int. 1.86 ms	12.2	473	0.078 s	[91]
		Rate: ~5 kHz					
		D: Stationary					
TSUNAMI	16.2/16.7/35.1	Sample: FNP	Piezo: ~1 ms Int.: 1 ms Offline: 50 μs	7.5	-	-	[92]
		Rate: ~100 kHz					
		Sample speed: 6 µm2/s					
3D-DyPLoT	6.6/8.7/15.6	Sample: FNP	Int.: 20 μs Piezo: ~1 ms	20	20	30 s for particle with 8.9 μm^2^/s dynamics	[93]
		Rate: ~80 kHz					
		D: Stationary					
Image-based (bi-plane)	7/8/27	Sample: FNP	Int.: 0.3 ms Piezo: 2 ms	2.4	3-4	3 s	[100]
		Rate:					
		D: Stationary					
Image-based (light-sheet)	10/10/40	Sample: FNP	Int.: 1.1 ms	9.4	-	-	[101]
		Rate: 750 photons					
		D: Stationary					

**Table 2:** Comparison of environmental contextualization metrics in active feedback RT-3D-SPT methods. Tracking temporal resolution derived from shortest integration time reported. Imaging time refers to the 2D frame time.

Method	Tracking Temporal Resolution	Imaging Time	Simultaneous Imaging	Optical Sectioning	Reference
TSUNAMI	50 µs	0.7 s	NO	YES	[92]
Orbital	32 ms	~200 ms	YES	NO	[84]
Tetrahedral Detection	5 ms	40 ms	YES	YES	[102]
3D-MM	10 µs	1.0 s	YES	YES	[76]
